# Complete plastome sequence of *Thalictrum coreanum* (Ranunculaceae) and transfer of the *rpl32* gene to the nucleus in the ancestor of the subfamily Thalictroideae

**DOI:** 10.1186/s12870-015-0432-6

**Published:** 2015-02-05

**Authors:** Seongjun Park, Robert K Jansen, SeonJoo Park

**Affiliations:** Department of Integrative Biology, University of Texas at Austin, 1 University Station C0930, Austin, TX 78712 USA; Department of Life Sciences, Yeungnam University, Gyeongsan, 712-749 Korea; Department of Biological Science, King Abdulaziz University, Jeddah, 21589 Saudi Arabia

**Keywords:** Gene loss, *infA*, Intracellular gene transfer, Meadow-rue, Plastid genome, *rpl32*

## Abstract

**Background:**

Plastids originated from cyanobacteria and the majority of the ancestral genes were lost or functionally transferred to the nucleus after endosymbiosis. Comparative genomic investigations have shown that gene transfer from plastids to the nucleus is an ongoing evolutionary process but molecular evidence for recent functional gene transfers among seed plants have only been documented for the four genes *accD*, *infA*, *rpl22*, and *rpl32*.

**Results:**

The complete plastid genome of *Thalictrum coreanum*, the first from the subfamily Thalictroideae (Ranunculaceae), was sequenced and revealed the losses of two genes, *infA* and *rpl32*. The functional transfer of these two genes to the nucleus in *Thalictrum* was verified by examination of nuclear transcriptomes. A survey of the phylogenetic distribution of the *rpl32* loss was performed using 17 species of *Thalictrum* and representatives of related genera in the subfamily Thalictroideae. The plastid-encoded *rpl32* gene is likely nonfunctional in members of the subfamily Thalictroideae (*Aquilegia*, *Enemion*, *Isopyrum*, *Leptopyrum*, *Paraquilegia*, and *Semiaquilegia*) including 17 *Thalictrum* species due to the presence of indels that disrupt the reading frame. A nuclear-encoded *rpl32* with high sequence identity was identified in both *Thalictrum* and *Aquilegia.* The phylogenetic distribution of this gene loss/transfer and the high level of sequence similarity in transit peptides suggest a single transfer of the plastid-encoded *rpl32* to the nucleus in the ancestor of the subfamily Thalictroideae approximately 20–32 Mya.

**Conclusions:**

The genome sequence of *Thalictrum coreanum* provides valuable information for improving the understanding of the evolution of plastid genomes within Ranunculaceae and across angiosperms. *Thalictrum* is unusual among the three sequenced Ranunculaceae plastid genomes in the loss of two genes *infA* and *rpl32*, which have been functionally transferred to the nucleus. In the case of *rpl32* this represents the third documented independent transfer from the plastid to the nucleus with the other two transfers occurring in the unrelated angiosperm families Rhizophoraceae and Salicaceae. Furthermore, the transfer of *rpl32* provides additional molecular evidence for the monophyly of the subfamily Thalictroideae.

**Electronic supplementary material:**

The online version of this article (doi:10.1186/s12870-015-0432-6) contains supplementary material, which is available to authorized users.

## Background

Massive transfer of genes from the plastid to the nucleus occurred following the endosymbiotic origin of the plastid from cyanobacteria [1]. Photosynthetic land plant plastid genomes (plastomes) only encode 101–118 genes, most of which represent genetic system and photosynthetic genes [2,3]. A considerable number of organelle-targeted genes in the nucleus are translated in the cytosol and imported into the plastids and mitochondria where they perform essential functions. Many studies have revealed that gene transfer from organelles to the nucleus is an ongoing process [1,4], however subsequent molecular characterization of these events has been limited. Transferred plastid genes must obtain nuclear expression elements as well as transit peptides for import of gene products into the plastids [5,6]. Successful functional gene transfers from the plastid to the nucleus in seed plants have been documented for only four genes: *infA* in multiple lineages [7], *rpl22* in Fabaceae and Fagaceae [8,9], *rpl32* in Rhizophoraceae and Salicaceae [10,11] and *accD* in *Trifolium* [12,13]. Transferred plastid genes have either adopted a transit peptide from an existing nuclear gene or acquired a novel transit peptide [9,10,13]. In addition to functional gene transfers, movement of DNA fragments from the plastid to the nucleus is common among flowering plants (referred to as NUPTs; nuclear plastid DNA) [1,14], and the proportion of NUPTs differs considerably among species [15,16].

The angiosperm family Ranunculaceae (buttercups) exhibits enormous ecological, anatomical, biochemical, and morphological diversity and comprises approximately 2,500 species in 59 genera and five subfamilies distributed throughout the world [17]. Ranunculaceae have two chromosome types: R (*Ranunculus*)-type with large chromosomes, and T (*Thalictrum*)-type with small chromosomes [17,18]. Although there are several different classification systems for Ranunculaceae [17,19-23], multiple lines of evidence suggest that genera with the T-type chromosome (excluding *Hydrastis*) form a monophyletic group [22-24]. *Thalictrum*, a member of the subfamily Thalictroideae, is one of the most diverse genera of Ranunculaceae in terms of number of species and morphological variation [17]. Recent studies have estimated phylogenetic relationships of *Thalictrum* using molecular data to understand the evolution of sexual systems and polyploidy [25,26]. This genus has great medicinal value because it contains high levels of Thaliblastin (Thalicarpine), which has anticancer properties [27,28]. *Thalictrum coreanum* is a popular, economically important endemic plant native to Korea and it is used widely in horticulture and medicine. Its natural habitat is restricted to small areas in Korea and it is often confused with a species of Berberidaceae, *Epimedium koreanum*, which is used in traditional Chinese and Korean herbal medicine as a potent enhancer of erectile function.

Previous studies performed restriction site mapping of the plastid genome of Ranunculaceae and identified several phylogenetically informative rearrangements, including inversions, the loss of the *rps16* gene and loss of the *rps12 cis*-spliced intron [29,30]. The complete plastid genome sequences of only two species of Ranunculaceae have been reported [31,32] and neither of these are members of the subfamily Thalictroideae.

In this study the plastome sequence of *T. coreanum* is presented, which represents the first sequenced member of the subfamily Thalictroideae. Genome organization is examined, including identification of transfers of two genes, *infA* and *rpl32*, from the plastid to the nucleus. In addition, the phylogenetic distribution of the *rpl32* gene loss in the Ranunculaceae is examined. The plastome sequence of *T. coreanum* provides valuable additional information about variation within the Ranunculaceae.

## Results

### Plastome of *Thalictrum coreanum*

**Figure 1:**
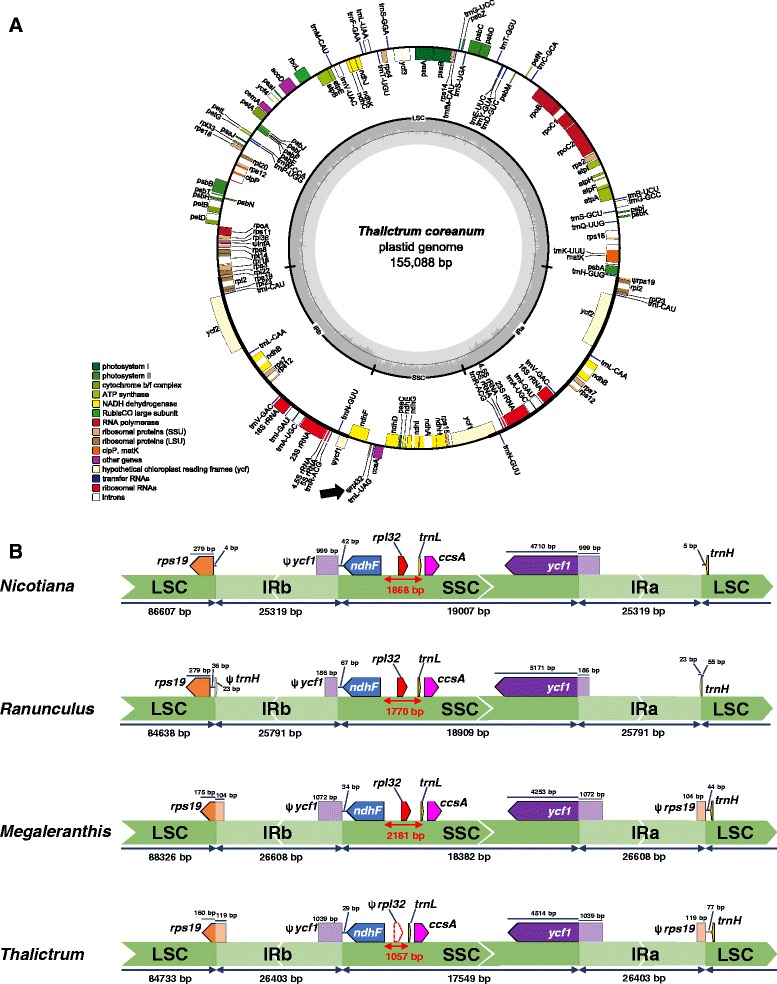
**Circular gene map of** ***Thalictrum coreanum*** **plastome (A) and comparison of inverted repeat region of three plastomes from Ranunculaceae (B). A**. Thick lines on inner circle indicate the inverted repeats (IRa and IRb, 26,403 bp), which separate the genome into small (SSC, 17,549 bp) and large (LSC, 84,733) single copy regions. Genes on the inside and outside of each map are transcribed clockwise and counterclockwise direction, respectively. The ring of bar graphs on the inner circle display GC content in dark grey. Ψ denotes a pseudogene and an arrow indicates the position of *rpl32* pseudogene. **B**. Inverted repeat (IR) boundaries in three Ranunculaceae plastid genomes with *Nicotiana tabacum* as a reference genome are highlighted. Lengths of genes, large single copy (LSC), small single copy (SSC), and IRs are not to scale.

The *Thalictrum coreanum* plastome is 155,088 bp with a pair of inverted repeats (IRs) of 26,403 bp separated by a small single copy (SSC) region of 17,549 bp and a large single copy (LSC) region of 84,733 bp (Figure 1A and Table 1). The genome encodes 112 different genes, including 78 protein-coding genes, 30 tRNA genes, and 4 rRNA genes and consists of 58.23% genes (i.e. protein-coding, tRNA, and rRNA genes) (Table 1). The translation initiation factor A (*infA*) is a pseudogene due to the presence of frameshift mutations. The ribosomal protein L32 (*rpl32*), which is usually located between *ndhF* and *trnL-UAG* (Figure 1A), is a pseudogene because deletions near the 5’ end generate two internal stop codons.

**Table 1:** **Comparison of Ranunculaceae plastome organization**

	***Thalictrum coreanum***	***Megaleranthis saniculifolia***	***Ranunculus macranthus***
Size (bp)	155,088	159,924	155,129
LSC length (bp)	84,733	88,326	84,638
SSC length (bp)	17,549	18,382	18,909
IR length (bp)	26,403	26,608	25,791
Number of different genes	112	114	113
protein-coding genes (duplicated in IR)	78 (6)	80 (6)	79 (6)
tRNA genes (duplicated in IR)	30 (7)	30 (7)	30 (7)
rRNA genes (duplicated in IR)	4 (4)	4 (4)	4 (4)
introns (duplicated in IR)	19 (5)	19 (5)	19 (5)
Percent of genome coding for genes (%)	58.23	56.52	58.18
Gene density*	0.82	0.81	0.83
Repetitive DNA (bp) (%)	187 (0.12)	545 (0.34)	283 (0.18)
GC content (%)	38.4	38.0	37.9

*Gene density indicates total number of genes/genome length including IR (genes/kb).

General features of the plastomes of three Ranunculaceae are summarized in Table 1. Compared with two other sequenced Ranunculaceae plastomes [31,32], *Megaleranthis saniculifolia* and *Ranunculus macranthus*, changes in genome organization reflect shifts of the IRs at the LSC/IR boundary relative to *Nicotiana tabacum* (Figure 1B). For example, IRb of *T. coreanum* and *M. saniculifolia* extends into the LSC to include the N-terminal portion of *rps19*, generating a truncated *rps19* fragment in IRa. However, in *R. macranthus*, IRa extends into the LSC to include the C-terminal portion of *trnH-GUG*, generating a *trnH-GUG* fragment in IRb. In terms of gene losses, the *infA* loss is shared by *T. coreanum* and *R. macranthus*, whereas *M. saniculifolia* contains an intact *infA* gene in its plastome. The presence of *rpl32* as a pseudogene is unique to *T. coreanum* among all three Ranuculaceae analyzed.

### Identification of functional gene transfers to the nucleus

**Figure 2:**
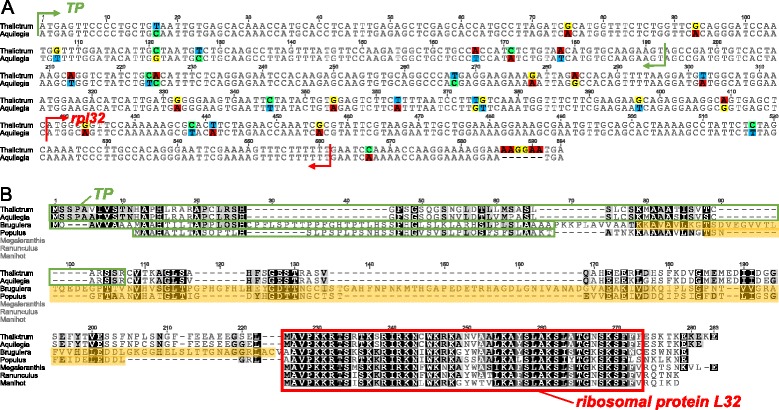
**Alignment of the ribosomal protein L32 gene** ***.*** **A**. Nucleotide sequence alignment of the nuclear-encoded *rpl32* copies from *Thalictrum* and *Aquilegia*. **B**. Amino acid sequence alignment of the nuclear copies of *rpl32* of *Thalictrum, Aquilegia,* and *Populus* with three plastid-encoded copies from related species. Green boxes indicate plastid transit peptides (TP) that were predicted using TargetP. Red box indicates a conserved domain of ribosomal protein L32. The shaded orange box indicates the putative Cu-Zn superoxide dismutase gene sequence.

To determine if the plastid-encoded *rpl32* gene in *Thalictrum* has been transferred to the nucleus, the transcriptome database (1KP project) for *T. thalictroides* was queried with the *rpl32* coding sequence of *M. saniculifolia* and *R. macranthus*. A transcript with high sequence identity to *rpl32* is present and has an extended sequence of 417 bp upstream from the conserved ribosomal protein L32 domain (CHL00152). The first 66 amino acids of the open reading frame (ORF) is predicted by both TargetP and Predotar to be a transit peptide that is targeted to the plastid (Table 2). The extended region including the transit peptide had no significant hits with BlastN to any sequences in the NCBI databases and Phytozome genomics portal. Extensive searching of the Phytozome genomics portal revealed the presence of a nuclear-encoded *rpl32* ORF in *Aquilegia coerulea*, which is also a member of the subfamily Thalictroideae. The sequence upstream from the conserved domain also has a transit peptide (66 amino acids; Table 2). However, an *rpl32*-like gene sequence was not detected in the *Hydrastis canadensis* transcriptome. Alignment of the nuclear-encoded *rpl32* from *Thalictrum* and *Aquilegia* revealed a pairwise nucleotide sequence identity of 94.2% and 93.2% for the extended region and the conserved domain, respectively (Figure 2A). Amino acid alignment of four nuclear-encoded *rpl32* copies (*Aquilegia*, *Thalictrum, Populus* [AB302219], and *Bruguiera* [AM711843]) shows that the extended region of *Thalictrum* is highly similar to *Aquilegia* with 89.9% identity, whereas *Populus* and *Bruguiera* are highly divergent with very low identities (19.3% and 16.5%) to *Thalictrum* (Figure 2B). The conserved ribosomal protein L32 domain of nuclear and plastid copies has pairwise identities ranging from 61.4% to 100% (Figure 2B).

**Table 2:** **Transit peptide prediction scores of putative nuclear-encoded plastid genes**

	**Predotar**		**TargetP**			
	**cTP**	**mTP**	**cTP**	**mTP**	**RC**	**Tplen**
*infA*†	**0.77**	0.01	**0.96**	0.02	1	59
infA*	**0.33**	0.01	**0.96**	0.07	1	58
*rpl32*†	**0.88**	0.01	**0.92**	0.07	1	66
rpl32*	**0.89**	0.01	**0.93**	0.08	1	66

cTP = chloroplast transit peptide. mTP = a mitochondrial targeting peptide. RC indicates reliability class, from 1 to 5, where 1 indicates the strongest prediction. Tplen means predicted presequence length (cleavage sites). Bold font indicates prediction of localization (chloroplast or mitochondrion). The symbols indicate the nuclear encoded *infA* (Aquca_001_00387.1; GBVZ2006252) and *rpl32* (Aquca_077_00029.1; GBVZ2008357) from *Aquilegia coerulea and †*Thalictrum thalictroides*, respectively*.*

Phylogenetic analyses of the nuclear-encoded *rpl32* copies (*Aquilegia*, *Bruguiera*, *Thalictrum*, and *Populus*) and the plastid-encoded copies from 48 other angiosperms show that the *Thalictrum* and *Aquilegia* nuclear copies are nested within a clade with the plastid copies of the two Ranunculaceae *Ranunculus* and *Megaleranthis*, and the *Populus* and *Bruguiera* nuclear-encoded copies are grouped with the rosid *Cucumis* (Additional file 1: Figure S1). The nuclear copies of *Thalictrum* and *Aquilegia* group together with high bootstrap support (100%). The branch lengths on the tree indicate that the four nuclear-encoded copies have much higher substitution rates compared to plastid-encoded copies of closely related species. However, bootstrap support across the angiosperms is weak because the tree is based on only a single, short gene sequence.

**Figure 3:**
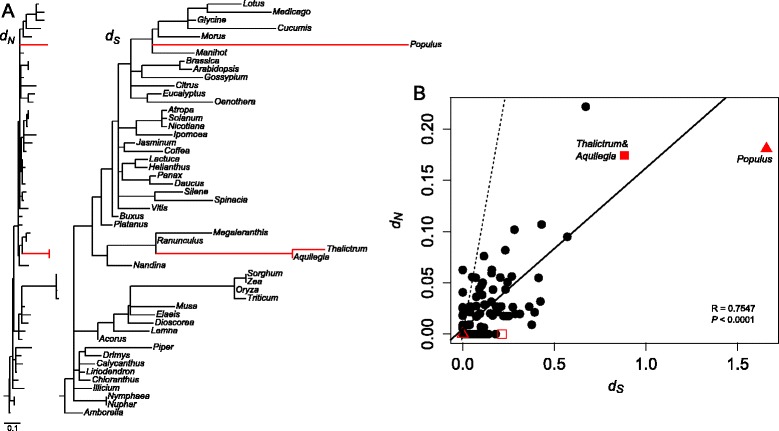
**Nuclear- and plastid-encoded** ***rpl32*** **divergence among selected angiosperms. A**. Maximum likelihood trees showing nonsynonymous (*d*_*N*_) and synonymous (*d*_*S*_) substitution rates for plastid-encoded *rpl32* genes with three nuclear-encoded copies. Red branches indicate the nuclear-encoded *rpl32* copies. Trees are drawn to the same scale shown in the bottom left. **B**. Correlation of synonymous and nonsynonymous substitution rates of *rpl32*. Significance of fit was evaluated by a Pearson correlation coefficient in the R package. The solid line represents the regression, which was analyzed using *d*_*N*_ and *d*_*S*_ on all branches except for the *Thalictrum* (open square), *Aquilegia* (open triangle), *Populus* (triangle) terminal branches, and the branch leading to *Thalictrum* and *Aquilegia* (square). The dashed line indicates *d*_*N*_/*d*_*S*_ ratio is equal to one.

To examine rate variation further, pairwise analysis of nonsynonymous (*d*_*N*_) and synonymous (*d*_*S*_) substitutions for plastid and nuclear *rpl32* homologs was performed (Figure 3). The analysis shows higher divergence in both *Aquilegia* and *Thalictrum* nuclear-encoded genes compared to other species of Ranunculaceae. Higher sequence divergence in the *Populus* nuclear-encoded copy is also evident. The synonymous substitution rate of *Thalictrum* and *Aquilegia* clade is 2.5 and 8.8 times higher than their closest relatives *Megaleranthis* and *Ranunculus*, respectively. The branch lengths on the tree indicate that the *Thalictrum* copy has experienced much higher synonymous substitution rates than *Aquilegia* (Figure 3A). The correlation of *d*_*N*_ and *d*_*S*_ was moderate (*P* < 1 x 10^−15^, *r* = 0.7547). The *d*_*N*_/*d*_*S*_ ratio among plastid copies shows similar patterns with *d*_*S*_ larger than *d*_*N*_, which is also the case for the three nuclear copies (Figure 3B).

In addition, a Blast search of the *T. thalictroides* transcriptome from the 1KP database identified one or more transcripts of the translation initiation factor IF1 (cd04451) domain that has a transit peptide for targeting back to the plastid (Table 2). The *Aquilegia* transcriptome databases from Phytozome v.10 were queried with the *infA* domain sequence from the *Thalictrum* nuclear copy, confirming an *infA*-like ORF acquired a transit peptide (Table 2). Examination of the *Aquilegia coerulea* v1.1 nuclear genome (Phytozome; scaffold_1) showed the presence of the nuclear-encoded *infA* gene containing two exons totaling 1,171 bp separated by a 105 bp intron (Additional file 1: Figure S2). Nuclear-like *infA* sequences were not detected in the *Hydrastis* transcriptome.

### Characterization of *rpl32* gene in the subfamily Thalictroideae

**Figure 4:**
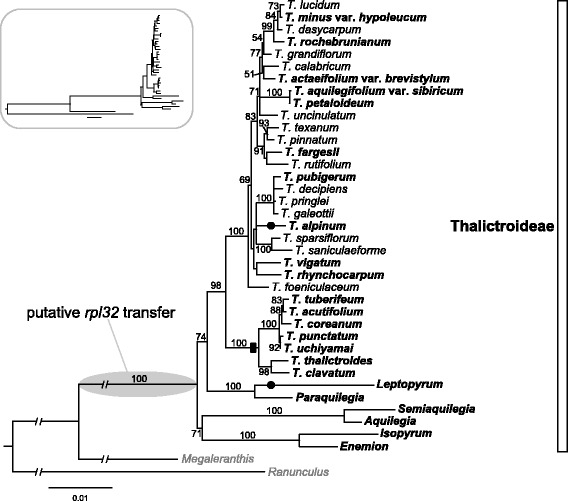
**Phylogenetic relationships among 37 species of the subfamily Thalictroideae.** Tree was constructed using nucleotide sequence of five plastid genes/regions (*rbcL*, *ndhF*, *ndhA* intron, *trnL* intron, and *trnL-F* intergenic spacer). The gray ellipse on node indicates putative transfer of *rpl32* to the nucleus and black dots indicate the complete loss of *rpl32* from plastid. Black rectangle on node indicates an indel event that is shared by members of the *T. coreaum* clade. Species in bold are those surveyed for loss of *rpl32*. Bootstrap support values > 50% are shown at nodes. Tree in box shows the original ML tree, which is broken (-//-) in the tree on right to make it easier to visualize. The circumscription of the subfamily Thalictroideae follows Wang et al. [23].

**Figure 5:**
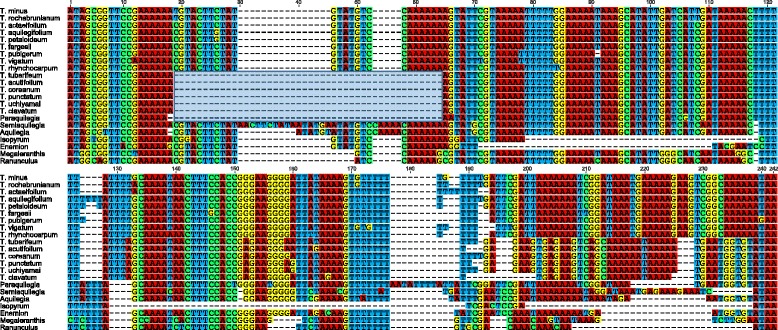
**Nucleotide alignment of** ***rpl32*** **gene/pseudogenes for Ranunculaceae.** The top 15 sequences represent putative *rpl32* pseudogenes for 15 *Thalictrum* species, the next five sequences are other genera within the subfamily Thalictroideae, and the bottom two sequences are representative species from outside of the subfamily Thalictroideae. Blue box shows an indel event that is shared by members of the *T. coreaum* clade.

The plastid-encoded *rpl32* is a pseudogene in *T. coreanum* (Figure 1A). Seventeen additional species of *Thalictrum* representing two subgenera were surveyed for the presence of a pseudogene using PCR and Sanger sequencing (Figure 4). In *T. thalictroides*, PCR failed to amplify a product, which may be due to variation in primer binding sites. The product sequence sizes for the other 16 species of *Thalictrum* range from 745 bp in *T. alpinum* to 1,198 bp in *T. rochebrunianum* (median size of 16 *Thalictrum* species was 1,104 bp). Blast searches using intact *rpl32* from *M. saniculifolia* (174 bp) and *R. macranthus* (162 bp) revealed that 15 examined *Thalictrum* species have remnant sequences of *rpl32*, ranging from 164 to 210 bp (Figure 5). However, one species, *T. alpinum*, lacks any detectable *rpl32*-like sequences, suggesting a loss of the entire gene. Nucleotide alignment of *rpl32* revealed a consistent pattern, the majority of indel events are shared by members of the *T. coreaum* clade (Figures 4 and 5).

To further investigate the *rpl32* gene loss, six other genera (*Aquilegia*, *Enemion*, *Isopyrum*, *Leptopyrum*, *Paraquilegia*, and *Semiaquilegia*) were examined in the subfamily Thalictroideae. The results show frameshift mutations due to insertions and deletions (indels) in five of the genera (Figure 5), and the sixth genus *Leptopyrum* has entirely lost *rpl32*. Maximum likelihood (ML) analysis of a concatenated data set resolves phylogenetic relationships among members of the subfamily Thalictroideae with bootstrap values of 98% for the monophyly of *Thalictrum* and 100% for the monophyly of subfamily Thalictroideae (Figure 4). Overall the *rpl32* gene in the plastid genome of subfamily Thalictroideae is likely nonfunctional due to indels that disrupt the reading frame.

### Correlation between reduction of *ndhF-trnL* intergenic spacer and *rpl32* gene loss

**Figure 6:**
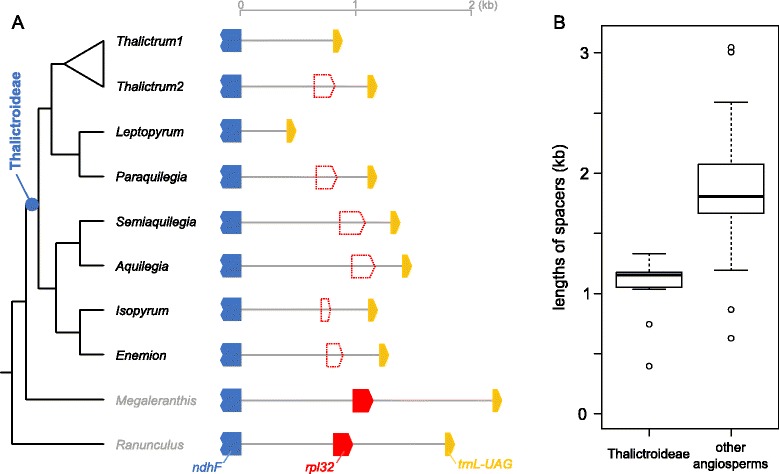
**Length variation of intergenic spacer including** ***rpl32*** **among species in the subfamily Thalictroideae. A**. Schematic diagram of the regions surrounding the *rpl32* gene in 22 sequenced species (right). In tree on left (reduced version of Figure 2), *Thalictrum1* indicates *Thalictrum alpinum* and *Thalictrum2* represents the remaining *Thalictrum* species. Dotted red boxes indicate the proportion of the remnant sequences from *rpl32*. **B**. Boxplot distribution of the lengths of the *ndhF*-*trnL* intergenic spacers between the subfamily Thalictroideae and other angiosperms that contain *rpl32* gene (Additional file 2: Table S3).

The *ndhF-trnL* intergenic spacer (IGS) including *rpl32* gene, which is either a pseudogene or absent within the subfamily Thalictroideae, shows considerable length variation (1.6-5.5 fold reduction compared to a full length IGS with *rpl32*, Figure 6A). This IGS region in the subfamily Thalictroideae is nearly two times shorter than in other angiosperms (Figure 6B). Both *t*-test and Wilcoxon signed rank test estimates indicated that the mean size of IGS between the two groups is significantly different (*t*-test; *P* < 1 × 10^−13^ and Wilcoxon signed-rank test; *P* < 1 × 10^−8^).

## Discussion

### Functional gene transfer to the nucleus

Two protein coding genes, translation initiation factor A (*infA*) and ribosomal protein L32 (*rpl32*), are pseudogenes in the *T. coreanum* plastome. In case of *infA*, multiple independent losses have been reported across angiosperms including *Caltha* from the Ranunculaceae [7,33]. This previous report, combined with the phylogenetic distribution of *infA* loss from the sequenced Ranunculaceae genomes, indicates that this gene has been lost multiple times in the family. In order for a gene transfer event to be successful, transferred genes must acquire a transit peptide to shuttle the product back into plastids. Nuclear-encoded *infA* copies from *Thalictrum* and *Aquilegia* were identified in the transcriptome and they have high levels of sequence identity. In view of the high nucleotide sequence identity of both *infA* (94.1%) and the transit peptide (85.1%), it is likely that there has been a single transfer of this gene to the nucleus within the subfamily Thalictroideae, although expanded sampling is needed to confirm this hypothesis.

Most ribosomal protein subunits have been transferred to the nuclear genome since the endosymbiotic origin of plastids; however, land plant plastid genomes still retain a set of 12 small ribosomal protein subunits (*rps*) and 9 large ribosomal protein subunits (*rpl*) [2]. Among the remaining plastid-encoded *rps* and *rpl* genes, several examples of gene losses across seed plants have been demonstrated [3,33]. Comparative analysis of the three sequenced Ranunculaceae plastomes (*Megaleranthis*, *Ranunculus*, and *Thalictrum*) indicates that the loss of the plastid-encoded *rpl32* gene is unique to the *Thalictrum* plastome. However, comparisons of 17 additional *Thalictrum* species suggest that pseudogenization of the plastid encoded *rpl32* gene occurred within the entire genus (Figure 4). Alignment of *rpl32* pseudogenes from the sequenced *Thalictrum* species with intact *rpl32* genes from *M. saniculifolia* and *R. macranthus* reveals that the majority of indel events are shared by members of the *T. coreaum* clade (Figure 5), indicating that the deletions occurred in the ancestor of this clade. Examination of the transcriptome sequences of *Thalictrum* and *Aquilegia* reveals that *rpl32* has been transferred to the nucleus and acquired a target peptide for transport back to the plastid (Figure 2). The nuclear copies from *Aquilegia* and *Thalictrum* have high sequence identity at both nucleotide and amino acid levels (93.9% and 92.8%). The transferred genes have significantly elevated synonymous substitution rates and have experienced purifying selection (Figure 3). Phylogenetic analysis provided strong support for monophyly of the nuclear-encoded *rpl32* copies (Figure 4), suggesting a single transfer of *rpl32* to the nucleus. Plastid-encoded *rpl32* gene losses have also been reported from *Bruguiera, Populus, Yucca,* and some parasitic plants [33,34]. There is evidence in only two of these cases, *Bruguiera* and *Populus*, that *rpl32* has been functionally transferred to the nucleus [10,11]. In the case of *Bruguiera* and *Populus rpl32* fused to an existing nuclear gene (Cu-Zn superoxide dismutase) to acquire a transit peptide, whereas *Thalictrum* and *Aquilegia* have acquired a novel transit peptide.

### Loss of plastid-encoded *rpl32* gene in the subfamily Thalictroideae

The high level of conservation of genome organization among the three sequenced Ranunculaceae plastomes enabled a PCR and sequencing survey of the *ndhF* and *trnL-UAG* region, which contains the *rpl32* gene. The absence of intact *rpl32* gene was identified for seven genera of the subfamily Thalictroideae (*Aquilegia*, *Enemion*, *Isopyrum*, *Leptopyrum*, *Paraquilegia*, *Semiaquilegia*, and *Thalictrum*) and the evolutionary fate of the plastid-encoded *rpl32* differed among the genera or species examined; the gene is completely absent in *Leptopyrum* and *T. alpinum* and pseudogenes of varying length are present in the remaining species (Figure 6A). This suggests that *rpl32* was transferred to the nucleus in the ancestor of subfamily Thalictroideae. Previous studies have shown that reductions of IGS regions are caused by gene loss, which has led to a more compact genome [35,36]. Although most examined Thalictroideae have a portion of *rpl32* remaining, the *ndhF-trnL* intergenic spacer is significantly shorter in the subfamily Thalictroideae than in other angiosperms (Figure 6B) due to extreme degradation of the IGS. This finding indicates that the reduction of the *ndhF* and *trnL-UAG* IGS region is associated with the loss or pseudogenization of *rpl32*.

Two different types of chromosomes based on size have been characterized in Ranunculaceae, R-type and T-type [17,18]. The subfamilies Thalictroideae and Hydrastidoideae belong to T-type chromosome group, however, phylogenetic analyses have shown that these two subfamilies are polyphyletic [23,24]. The distribution of the transfer of *rpl32* to the nucleus in *Thalictrum* and *Aquilegia* but not in *Hydrastis* indicates that this transfer does not represent a synapomorphy for the lineages with the T-type chromosomes.

Fior et al. [37] used the *rbcL*, *matK* and 26S nuclear ribosomal DNA (nrDNA) sequences generated by Wang et al. [23] to infer divergence times for the main clades of the Ranunculaceae. The divergence time of the subfamily Thalictroideae was estimated at 26.2 Mya (95% highest posterior density, HPD = 20.3-32.3 Mya). Another estimate indicated slightly later divergence times with the shorter interval for the subfamily Thalictroideae at 27.61 Mya (95% HPD = 26.6-28.6 Mya) [38]. Thus, the transfer of *rpl32* to the nucleus at the base of the subfamily Thalictroideae occurred approximately 20–32 Mya.

The monophyly of subfamily Thalictroideae has been confirmed based on phylogenetic analyses of multiple DNA markers: *rbcL*, *matK*, *trnL-F* spacer, and 26S nrDNA [23], 26S nrDNA [24], and *atpB*, *rbcL*, and 18S nrDNA [39]. The *rpl32* gene transfer event, combined with divergence time estimates, provides valuable phylogenetic data in support of the monophyly of subfamily Thalictroideae. Although there are multiple examples of plastid gene losses that exhibit homoplasy [e.g., 7, 9], the loss of *rpl32* by all sampled members of subfamily Thalictroideae provides an excellent example of a genomic change that supports the monophyly of this subfamily.

## Conclusions

The plastome sequence of *Thalictrum coreanum,* the first genome completed from the subfamily Thalictroideae, provides new insights into the evolution of plastomes within Ranunculaceae. The *T. coreanum* plastome is highly conserved with gene order identical to the ancestral organization of angiosperms [40] and at 155 kb it has the median genome size for photosynthetic land plants [41]. The only unusual feature of the plastome is the loss of two genes, *infA* and *rpl32*. Examination of nuclear transcriptomes indicates that both of these genes have been transferred to the nucleus. Comparing the plastome sequence of *Thalictrum* with the two other Ranunculaceae and the survey of the *rpl32* gene loss resolve the phylogenetic distribution and timing of this gene loss/transfer event in Ranunculaceae.

## Methods

### Plant material, plastid isolation, and RCA

Fresh leaf tissue of *Thalictrum coreanum* was sampled from a single individual from a natural population in Gangwon-do, Korea. Intact plastids were isolated from 1.45 g of tissue using the sucrose step gradient method of Jansen et al. [42]. Isolated plastids were used to amplify the plastid genome by rolling circle amplification (RCA) using REPLI-g midi Kit (cat. No. 150043, Qiagen, Valencia, CA, USA) following the protocol described in Jansen et al. [42]. RCA products were digested with *EcoRI* and the resulting fragments were separated by gel electrophoresis in a 1% agarose gel to verify the purity and quantity of plastid DNA.

### Genome sequencing, assembly, annotation, and analyses

Plastid DNA (538.9 ng/ul) was sheared by nebulization, subjected to library preparation and sequencing on a Roche 454 Genome Sequencer (GS) FLX Titanium platform at Solgent Co. (Deajeon, Korea). The Roche 454 sequencing produced approximately 80 Mb of sequence with an average read length of 357 bp.

The quality filtered sequence reads were assembled using the GS *de novo* sequence assembler v.2.5.3 (Roche 454 Life Sciences, Branford, CT, USA) and multiple assemblies were performed with modified parameters (i.e. adjusting minimum overlap length). Three long contigs representing a nearly complete plastid genome sequence were generated and the contigs were mapped against two complete plastid genomes of Ranunculaceae, *Megaleranthis saniculifolia* (NC_012615) and *Ranunculus macranthus* (NC_008796), in Geneious R6 v.6.1.6 [43]. The presence of gaps between the junctions of LSC, SSC, and IR regions were filled by polymerase chain reaction (PCR) and Sanger sequencing. The Roche 454 pyrosequencing platform is known to have a high error rate in long homopolymer regions [44,45]. There were 36 homopolymers > 7 bp in protein-coding genes, five of which were nonsense mutations, and these regions were corrected by PCR and Sanger sequencing. All primers for PCR were designed by Primer3 [46] in Geneious R6 (Additional file 2: Table S1).

Annotation of plastid genome was done in DOGMA [47] and all tRNA genes were verified by their predicted secondary structures using tRNAscan-SE 1.3.1 [48]. A genome map was drawn with OGDRAW [49]. The plastome sequence of *T. coreanum* was deposited in GenBank (accession number KM206568).

Two published plastomes of Ranunculaceae [31,32], *M. saniculifolia* and *R. macranthus*, were used for genomic comparisons with *T. coreanum*. Whole genome alignment was performed under ‘progressiveMauve algorithm’ [50] in Geneious R6. Repetitive sequences were identified by performing BLASTN v.2.2.28+ (word size = 11) searches of each plastome against itself with an e-value cutoff of 1e^−10^ and at least 90% sequence identity. The analysis was performed on Lonestar Dell Linux Cluster of the Texas Advanced Computing Center (TACC).

### Identification of gene transfers to the nucleus

Three genera of Ranunculaceae (*Aquilegia*, *Hydrastis*, and *Thalictrum*) with T-type chromosomes were surveyed for gene transfers to the nucleus. *Thalictrum thalictroides* and *H. canadensis* transcriptomes from the 1KP project database [51] and *A. coerulea* transcriptome from the genomics portal Phytozome v.10 [52] were searched. Transferred genes were identified using BlastN of the *infA* and *rpl32* sequences from the *M. saniculifolia* and *R. macranthus* plastomes against the transcriptomes. The NCBI Conserved Domain Database (CDD) was used for functional domain annotation [53]. TargetP v.1.1 [54] and Predotar v.1.03 [55] were used to predict transit peptides. Putative ORFs were searched for using Phytozome with BLASTX and ‘angiosperms’ as a reference sequence source to identify plant gene families. Nucleotide and amino acid sequences of nuclear and plastid genes were aligned with MUSCLE [56] in Geneious R6.

### Survey for loss of *rpl32* gene in the subfamily Thalictroideae

Seventeen *Thalictrum* species from all major clades of the phylogenetic tree of the genus (S. Park, unpublished) and six other genera of the subfamily Thalictroideae were sampled (Additional file 2: Table S2). Total genomic DNA was isolated from either fresh leaves or herbarium specimens using the methods of Allen et al. [57] with the following modifications to the extraction buffer: Cetyl trimethylammonium bromide (CTAB) was increased to 3%; and 1% polyvinylpyrrolidone (PVP, w/v, MW 4,000) and 2% beta-mercaptoethanol (Sigma, St. Louis, MO) were added. To detect the *rpl32* gene, the intergenic spacer (IGS) region between *ndhF* and *trnL-UAG* genes was amplified by PCR using the Shaw et al. [58] primers (*ndhF*: GAAAGGTATKATCCAYGMATATT and *trnL-UAG*: CTGCTTCCTAAGAGCAGCGT). PCR products were purified by using Solg™ Gel & PCR Purification System Kit (Solgent Co., Daejeon, Korea) following the manufacturer’s protocol. All sequencing of PCR products was performed using an ABI 3730XL DNA Analyzer (Applied Biosystems, California, USA) at Solgent Co., and nucleotide sequences were aligned with MUSCLE in Geneious R6. Statistical analysis was conducted by using R v.2.1.5 [59] to test whether gene loss/transfer was associated with the size of intergenic spacer.

### Phylogenetic analyses

Phylogenetic analyses were performed on two data sets. The first included 39 species with nucleotide sequence of five plastid genes/regions (*rbcL*, *ndhF*, *ndhA* intron, *trnL* intron, and *trnL-F* intergenic spacer), including 31 *Thalictrum* species and a single species from six other genera of the subfamily Thalictroideae (Additional file 2: Table S2). *Megaleranthis saniculifolia* and *R. macranthus* were used as outgroups by extracting nucleotide sequences of the five genes/regions from the published plastomes. The second data set included sequences of the plastid-encoded *rpl32* gene for 48 taxa and four nuclear-encoded copies (Additional file 2: Table S3). The data sets were aligned with MUSCLE in Geneious R6. Maximum likelihood (ML) analyses were performed with RAxML v.7.2.8 [60] using the ‘GTRGAMMA’ model under the rapid bootstrap algorithm with 1000 replicates at TACC.

### Estimating nucleotide substitution rates

To analyze rates of nucleotide substitution, photosystem I (*psaA*, *B*, and *C*) and II (*psbB*, *C*, *D*, *E*, *F*, *H*, *J*, *L*, *M*, *N*, *T*, and *Z*) genes and *rbcL* were sampled from selected angiosperms (Additional file 2: Table S3). The data were concatenated into a single data set and a phylogenetic tree was generated using the ML method (see phylogenetic analyses section) and used as a constraint tree (Additional file 1: Figure S3) for all rate comparisons. Nonsynonymous (*d*_*N*_) and synonymous (*d*_*S*_) substitution rates for 48 plastid-encoded *rpl32* sequences and three nuclear-encoded sequences (*rpl32* from *Bruguiera* was not used in the rate variation estimation because there were insufficient plastid data to generate a constraint tree) were calculated in PAML v.4.8 [61] using codeml option with codon frequencies estimated with an F3 × 4 model.

### Availability of supporting data

The data sets supporting the results of this article are included within additional files. The phylogenetic data sets (including amino acid sequence) supporting the results of this article are available in Dryad Digital Repository (http://dx.doi.org/10.5061/dryad.g84g5).

## Additional files

Additional file 1: Figure S1. Maximum likelihood phylogenetic tree of 52 taxa based on *rpl32* gene sequence. **Figure S2.** Alignment of the *infA* gene. **Figure S3.** Maximum likelihood phylogenetic tree inferred from 16 genes from 51 taxa of angiosperms used as a constraint tree for rate comparisons. Additional file 2: Table S1. Primers used for amplification. **Table S2.** NCBI accession numbers for species included in the phylogenetic analysis based on five plastid regions. **Table S3.** Taxon sampling and NCBI accession numbers for phylogenetic analyses, estimating nucleotide substitution rates, and examination of the correlation between reduction of *ndhF-trnL* intergenic spacer and *rpl32* gene loss.

## Abbreviations

NUPTs: Nuclear DNAs of plastid origin
ORF: Open reading frame
*d*_*N*_: Number of substitutions per nonsynonymous site
*d*_*S*_: Number of substitutions per synonymous site
rRNA: Ribosomal RNA
tRNA: Transfer RNA
IGS: Intergenic spacer
RCA: Rolling circle amplification
Mya: Million years ago
ML: Maximum likelihood
TACC: Texas advanced computing center
