# Supplementary material for: Complete plastome sequence of Thalictrum coreanum (Ranunculaceae) and transfer of the rpl32 gene to the nucleus in the ancestor of the subfamily Thalictroideae
Source: BMC Plant Biol. 2015 Feb 5;15:40. doi: 10.1186/s12870-015-0432-6 (PMC4329224; doi:10.1186/s12870-015-0432-6)

Figure S1. Maximum likelihood phylogenetic tree of 52 taxa based on *rpl32* gene sequence. Bootstrap support values >50% are shown at nodes. Red and black lines indicate nuclear- and plastid-encoded copies of *rpl32* sequences, respectively. Scale bar indicates 0.05 substitutions/site.

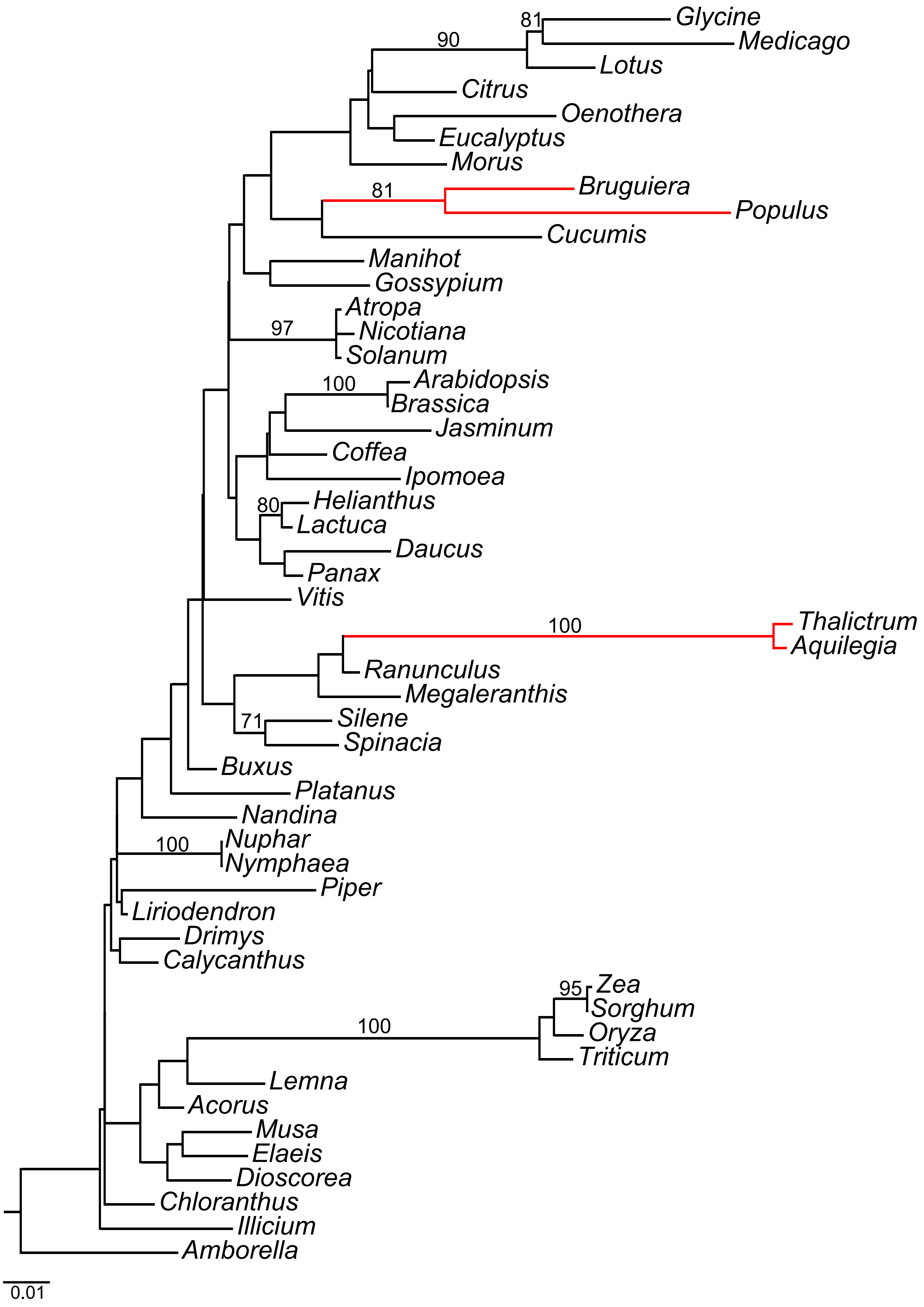

Figure S2. Alignment of the *infA* gene. **A.** Nucleotide sequence alignment of nuclear and transcript copies from *Aquilegia*. Intron is highlighted in colors. **B.** Amino acid sequence alignment of the nuclear copies of *rpl32* of *Thalictrum* and *Aquilegia*. Green box indicates plastid transit peptides (TP) that were predicted using TargetP. Red box indicates a conserved domain of translation initiation factor A.

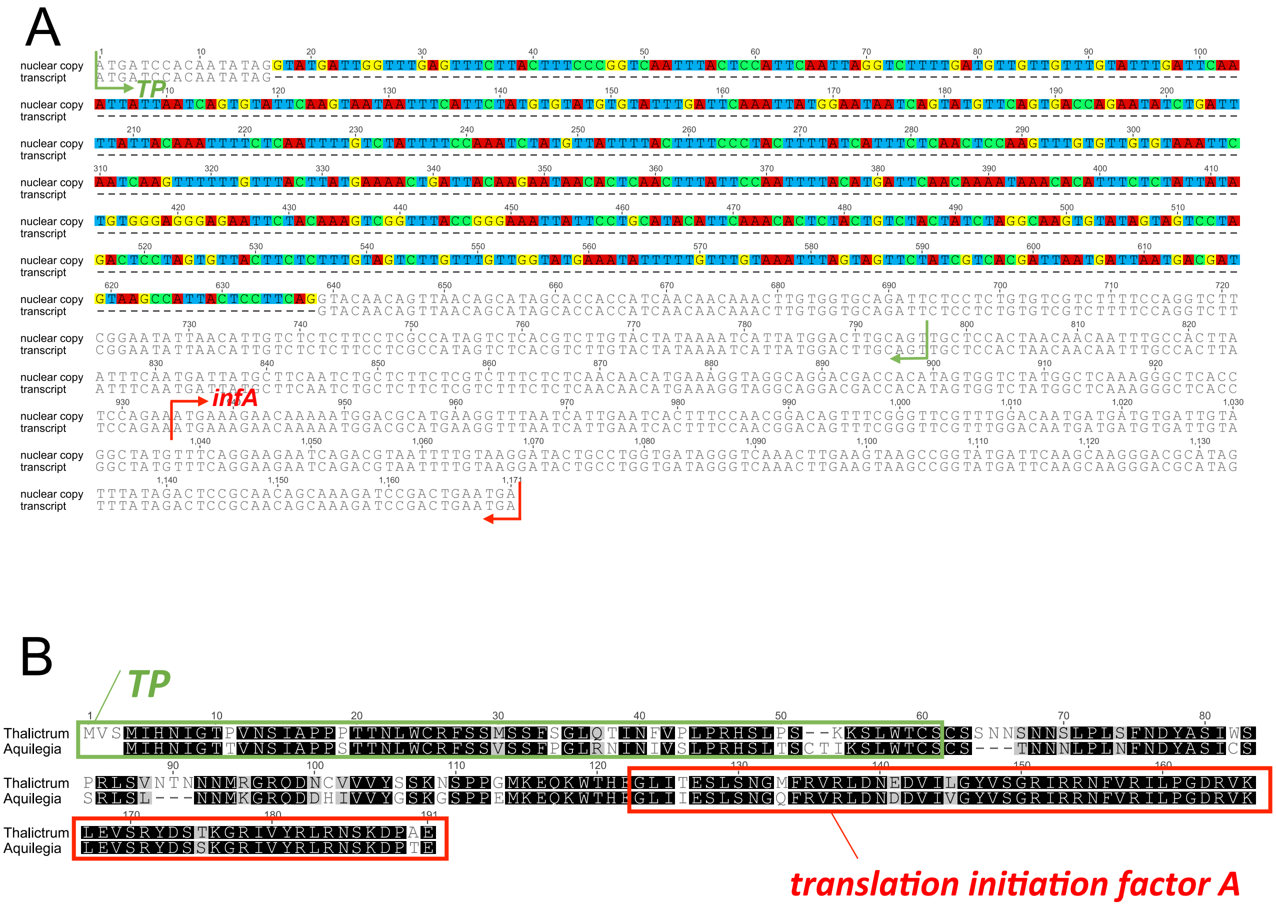

Figure S3. Maximum likelihood phylogenetic tree inferred from 16 genes from 51 taxa of angiosperms used as a constraint tree for rate comparisons. Bootstrap support values >50% are shown at nodes. Scale bar indicates 0.01 substitutions/site.

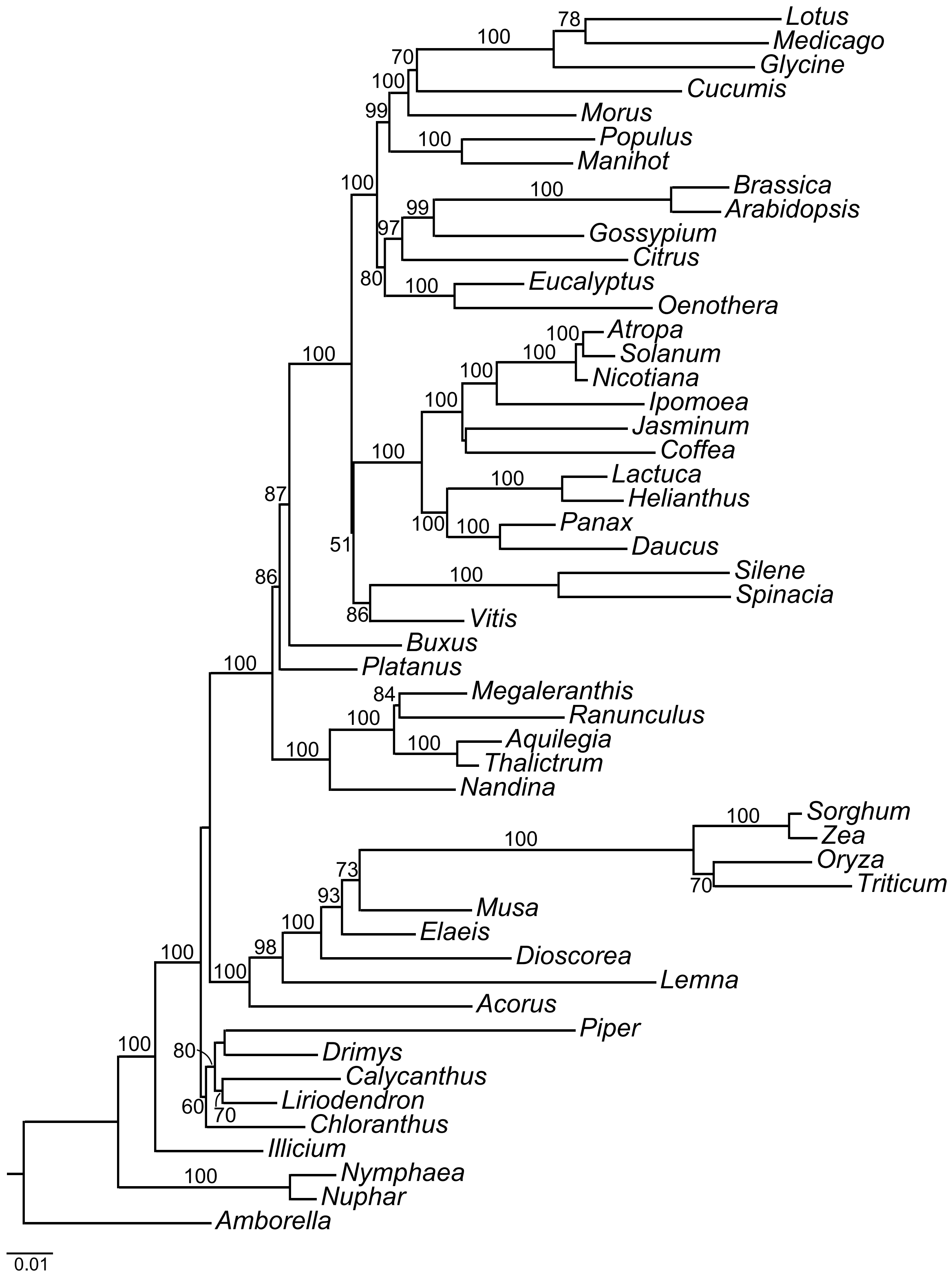

Supplement: Additional file 1: Figure S1. — Maximum likelihood phylogenetic tree of 52 taxa based on rpl32 gene sequence. Figure S2. Alignment of the infA gene. Figure S3. Maximum likelihood phylogenetic tree inferred from 16 genes from 51 taxa of angiosperms used as a constraint tree for rate comparisons. [file 12870_2015_432_MOESM1_ESM.pdf]
