# Supplementary material for: Complete plastome sequence of Thalictrum coreanum (Ranunculaceae) and transfer of the rpl32 gene to the nucleus in the ancestor of the subfamily Thalictroideae
Source: BMC Plant Biol. 2015 Feb 5;15:40. doi: 10.1186/s12870-015-0432-6 (PMC4329224; doi:10.1186/s12870-015-0432-6)
Supplement: Additional file 2: Table S1. — Primers used for amplification. Table S2. NCBI accession numbers for species included in the phylogenetic analysis based on five plastid regions. Table S3. Taxon sampling and NCBI accession numbers for phylogenetic analyses, estimating nucleotide substitution rates, and examination of the correlation between reduction of ndhF-trnL intergenic spacer and rpl32 gene loss. [file 12870_2015_432_MOESM2_ESM.pdf]

Table S1. Primers used for amplification.

	Primer name	Sequence (5' → 3')
Gap filling	LSC_IRaF	ACT CTT CGC GCT TTA TGA GCA GAC A
	LSC_IRaR	GCT CAG CAA CAG TCG GAC AAG TGG
	SSC_IRaF	CCC TTC CCC TTC CCT TTG TTC GGA
	SSC_IRaR	TGT AGG TGG AGG GCG CAT ATC TTC T
	SSC_IRbF	ACG TTG ATA GAC TGG GCG GGT ATG T
	SSC_IRbR	CCC TTC CCC TTC CCT TTG TTC GGA
	LSC_IRbF	GAT AGT TTG ATT CTT CGT CGC C
	LSC_IRbR	CGG ATG TAG CCA AGT GGA TC
Homopolymers	rpoC2F1	CCC GCC CGC TAT TTC ATA CGT GAC
	rpoC2F2	TCC CGG TGG TAT TAA GAT GCC GCT
	rpoC2R	CCC GAG GAA GTG CAT ATA TTA CCC GGA
	rpoBF	CCA CCT ATA GTT CCC CGA ACC GCA
	rpoBR	GGG CGC GTG TGA GTA GGA AAC AAA
	ndhFF	TTA ACC ATT TTG ATA ATA TAT CAA
	ndhFR	ATA AAA GCC AGA ACA TGG CTC TTA
	ycf1F	CGT CCC ACT CGG GGA CTT GAA AGA
	ycf2R	ACA ACT GCG CAT TCC CCG CTT TTT

Table S2. NCBI accession numbers for species included in the phylogenetic analysis based on five plastid regions.

Taxa	*rbcL*	*ndhF*	*ndhA* intron	*trnL* intron/ *trnL-F* IGS
T. actaeifolium var. *brevistylum* Nakai	JX258330	KM206570	JX258545	JX573433
T. alpinum L.	JX258333	KM206573	JX258548	JX573436
T. aquilegifolium var. *sibiricum* Regel & Tiling	JX258336	KM206576	JX258551	JX573439
T. calabricum Spreng.	JX258342	KM206581	JX258556	JX573443
T. clavatum DC.	JX258343	KM206582	JX258557	JX573444
T. coreanum H.Lév.	JX258346	KM206585	JX258560	JX573447
T. dasycarpum Fisch. & Avé-Lall.	JX258349	KM206588	JX258563	JX573450
T. decipiens B.Boivin	JX258351	KM206590	JX258565	JX573452
T. fargesii Franch. ex Finet & Gagnep.	JX258356	KM206595	JX258570	JX573457
T. foeniculaceum Bunge	JX258359	KM206598	JX258573	JX573460
T. galeottii Lecoy.	JX258362	KM206601	JX258576	JX573463
T. grandiflorum Maxim.	JX258364	KM206603	JX258578	JX573465
T. lucidum L.	JX258371	KM206610	JX258585	JX573472
T. minus var. *hypoleucum* (Siebold & Zucc.) Miq.	JX258373	KM206612	JX258587	JQ691515
T. petaloideum L.	JX258379	KM206618	JX258593	JX573479
T. pinnatum S.Watson	JX258380	KM206619	JX258594	JX573480
T. pringlei S.Watson	JX258384	KM206623	JX258598	JX573484
T. pubigerum Benth.	JX258389	KM206628	JX258603	JX573489
T. punctatum H.Lév.	JX258390	KM206629	JX258604	JQ691528
T. rhynchocarpum Quart.-Dill. & A.Rich.	JX258394	KM206633	JX258608	JX573493
T. rochebrunianum Franch. et Sav.	JX258395	KM206634	JX258609	JX573494
T. rutifolium Hook.f. & Thomson	JX258400	KM206639	JX258614	JX573499
T. saniculaeforme DC.	JX258402	KM206641	JX258616	JX573501
T. sparsiflorum Turcz.ex Fisch.& C.A.Mey.	JX258406	KM206644	JX258620	JQ691522
T. texanum (E.Hall ex A.Gray) Small	JX258411	KM206650	JX258625	JX573509
T. thalictroides (L.) A.J.Eames & B.Boivin	JX258413	KM206652	JX258627	JX573511
T. tuberifeum Maxim.	JX258415	KM206654	JX258629	JX573513
T. uchiyamai Nakai	JX258417	KM206656	JX258631	JX573515
T. uncinulatum Franch. ex Lecoy.	JX258419	KM206658	JX258633	JX573517
T. virgatum Hook.f. & Thomson	JX258422	KM206661	JX258636	JX573520
T. acutifolium (Hand.-Mazz.) B.Boivin	JX258423	KM206662	JX258637	JX573521
Enemion raddeanum Regal	JX258430	KM206669	JX258645	JQ691533
Isopyrum manshuricum Kom.	JX258431	KM206670	JX258646	JQ691532
Aquilegia buergeriana var. *oxysepala* (Trautv. & C.A.Mey.) Kitam.	JX258432	KM206671	JX258647	JQ691534
Semiaquilegia adoxoides (DC.) Makino	JX258433	KM206672	JX258648	JX573529
Paraquilegia microphylla (Royle) J.R.Drumm. & Hutch.	JX258434	KM206673	JX258649	JX573530
Leptopyrum fumarioides (L.) Reichb.	JX258435	KM206674	JX258650	JX573531

Table S3. Taxon sampling for phylogenetic analyses, estimating nucleotide substitution rates, and examination of the correlation between reduction of *ndhF-trnL* intergenic spacer and *rpl32* gene loss.

Groups	Order	Species	NCBI accession numbers
Basal Angiosperms	Amborellales	*Amborella trichopoda*	NC_005086
	Austrobaileyales	*Illicium oligandrum*	NC_009600
	Chloranthales	*Chloranthus spicatus*	NC_009598
	Nymphaeales	*Nuphar advena*	NC_008788
	Nymphaeales	*Nymphaea alba*	NC_006050
Magnoliids	Canellales	*Drimys granadensis*	NC_008456
	Laurales	*Calycanthus floridus*	NC_004993
	Magnoliales	*Liriodendron tulipifera*	NC_008326
	Piperales	*Piper coenoclatum*	NC_008457
Monocots	Acorales	*Acorus americanus*	NC_010093
	Arecales	*Elaeis guineensis*	NC_017602
	Dioscoreales	*Dioscorea elephantipes*	NC_009601
	Poales	*Oryza sativa*	NC_001320
	Poales	*Sorghum bicolor*	NC_008602
	Poales	*Triticum aestivum*	NC_002762
	Poales	*Zea mays*	NC_001666
	Zingiberales	*Musa textilis*	NC_022926
	Alismatales	*Lemna minor*	NC_010109
Eudicots	Apiales	*Daucus carota*	NC_008325
	Apiales	*Panax schinseng*	NC_006290
	Asterales	*Helianthus annuus*	NC_007977
	Asterales	*Lactuca sativa*	NC_007578
	Brassicales	*Arabidopsis thaliana*	NC_000932
	Brassicales	*Brassica rapa*	NC_015139
	Buxales	*Buxus microphylla*	NC_009599
	Caryophyllales	*Spinacia oleracea*	NC_002202
	Cucurbitales	*Cucumis sativus*	NC_007144
	Fabales	*Glycine max*	NC_007942
	Fabales	*Lotus corniculatus*	NC_002694
	Fabales	*Medicago truncatula*	NC_003119
	Gentianales	*Coffea arabica*	NC_008535
	Lamiales	*Jasminum nudiflorum*	NC_008407
	Malphigiales	*Bruguiera gymnorhiza*	- / AM711843*
	Malphigiales	*Manihot esculenta*	NC_010433
	Malphigiales	*Populus alba*	NC_008235 / AB302219*

Malvales	*Gossypium hirsutum*	NC_007944
Myrtales	*Eucalyptus globulus*	NC_008115
Myrtales	*Oenothera elata*	NC_002693
Proteales	*Platanus occidentalis*	NC_008335
Ranunculales	*Nandina domestica*	NC_008336
Ranunculales	*Ranunculus macranthus*	NC_008796
Ranunculales	*Megaleranthis saniculifolia*	NC_012615
Ranunculales	*Thalictrum coreaum*	KM206568 / [a]GBVZ[2008357]
Ranunculales	*Aquilegia coerulea*	AC253869
Rosales	*Morus indica*	NC_008359
Sapindales	*Citrus sinensis*	NC_008334
Solanales	*Atropa belladonna*	NC_004561
Solanales	*Ipomoea purpurea*	NC_009808
Solanales	*Nicotiana tabacum*	NC_001879
Solanales	*Solanum tuberosum*	NC_008096
Vitales	*Vitis vinifera*	NC_007957
Caryophyllales	*Silene latifolia*	NC_016730

Asterisk indicates the nuclear encoded *rpl32* genes. [a]The 1000 plants (1KP) database was searched using Blast for identifying the nuclear-encoded *rpl32* gene of *Thalictrum thalictroides*.
